# Downregulation of miR‐100‐5p in cancer‐associated fibroblast‐derived exosomes facilitates lymphangiogenesis in esophageal squamous cell carcinoma

**DOI:** 10.1002/cam4.6078

**Published:** 2023-05-15

**Authors:** Chao Chen, Chenbo Yang, Xiangyu Tian, Yinghao Liang, Shuaiyuan Wang, Xiaoqian Wang, Yuwei Shou, Hui Li, Qiankun Xiao, Jiao Shu, Miaomiao Sun, Kuisheng Chen

**Affiliations:** ^1^ School of Basic Medical Sciences Zhengzhou University Zhengzhou China; ^2^ Henan Key Laboratory of Tumor Pathology Zhengzhou University Zhengzhou China; ^3^ Department of Neurosurgery The Fifth Affiliated Hospital of Zhengzhou University Zhengzhou China; ^4^ Department of Pathology The First Affiliated Hospital of Zhengzhou University Zhengzhou China; ^5^ Department of Osteology The First Affiliated Hospital of Zhengzhou University Zhengzhou China; ^6^ BGI College & Henan Institute of Medical and Pharmaceutical Sciences Zhengzhou University Zhengzhou China

**Keywords:** cancer‐associated fibroblasts, esophageal squamous cell carcinoma, exosomes, lymphangiogenesis, miR‐100‐5p

## Abstract

**Background:**

Esophageal squamous cell carcinoma (ESCC), an aggressive gastrointestinal tumor, often has high early lymphatic metastatic potential. Cancer‐associated fibroblasts (CAFs) are primary components in tumor microenvironment (TME), and the impact of CAFs and its derived exosomes on lymphangiogenesis remains elusive.

**Materials and Methods:**

CAFs and the microlymphatic vessel density (MLVD) in ESCC was examined. Exosomes were extracted from primary normal fibroblast (NFs) and CAFs. Subsequently, tumor‐associated lymphatic endothelial cells (TLECs) were treated with these exosomes, and the effect on their biological behavior was examined. miR‐100‐5p was selected as the target miRNA, and its effect on TLECs was examined. The target of miR‐100‐5p was predicted and confirmed. Subsequently, IGF1R, PI3K, AKT, and p‐AKT expression in TLECs and tumors treated with exosomes and miR‐100‐5p were examined.

**Results:**

A large number of CAFs and microlymphatic vessels were present in ESCC, leading to a poor prognosis. CAF‐derived exosomes promoted proliferation, migration, invasion, and tube formation in TLECs. Further, they also enhanced lymphangiogenesis in ESCC xenografts. miR‐100‐5p levels were significantly lower in CAF‐derived exosomes than in NF‐derived exosomes. miR‐100‐5p inhibited proliferation, migration, invasion, and tube formation in TLECs. Further, miR‐100‐5p inhibited lymphangiogenesis in ESCC xenografts. Mechanistic studies revealed that this inhibition was mediated by the miR‐100‐5p‐induced inhibition of IGF1R/PI3K/AKT axis.

**Conclusion:**

Taken together, our study demonstrates that CAF‐derived exosomes with decreased miR‐100‐5p levels exhibit pro‐lymphangiogenesis capacity, suggesting a possibility of targeting IGF1R/PI3K/AKT axis as a strategy to inhibit lymphatic metastasis in ESCC.

## Graphical abstract

(1) CAFs affect the progression of ESCC by promoting lymphangiogenesis. (2) CAF‐derived exosomes promote the proliferation, invasion, migration and tube formation of TLECs. (3) CAF‐derived exosomal miR‐100‐5p affects ESCC lymphangiogenesis through the IGF1R/PI3K/ AKT pathway.

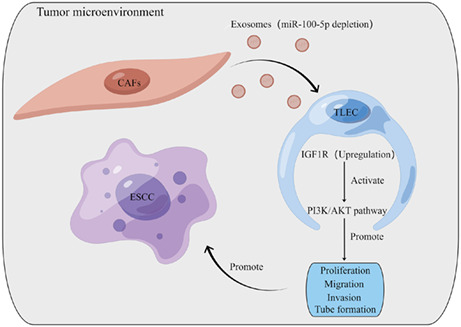

## 1 INTRODUCTION

Esophageal cancer is a tumor originating from esophageal mucosal epithelial cells or glandular epithelial cells. According to the GLOBOCAN 2020 study published by the International Cancer Research Agency, 604,000 people were newly diagnosed with esophageal cancer in 2020 and 544,000 people died from the disease. Accordingly, esophageal cancer had the seventh and sixth highest morbidity and mortality among all malignancies, respectively.^1^ The histological types of esophageal cancer are mainly divided into esophageal adenocarcinoma (EAC), which is the main type of esophageal cancer cases in developed countries, and esophageal squamous cell carcinoma (ESCC), which is the main type of esophageal cancer cases in developing countries, including China.^2^ The most common type of ESCC, usually shows metastasis—especially lymphatic metastasis—during the early stage of cancer development. Lymphangiogenesis is involved in lymphatic metastasis of ESCC, which leads to a poor prognosis of patients.^3^, ^4^ Our team found that the lymphatic endothelial cells can promote the proliferation and invasion of ESCC in vitro and in vivo.^5^ Therefore, there is a dire need to understand potential mechanisms of ESCC lymphangiogenesis.

Cancer‐associated fibroblasts (CAFs) make up the main component of tumor stroma in the tumor microenvironment (TME). CAFs not only serve as a structural skeleton, providing mechanical support to tumor cells, but also promote the occurrence and development of tumors through paracrine pathways, exosomal pathways, immune response regulation pathways, and extracellular stromal remodeling.^6^, ^7^ CAFs can contribute to the occurrence, growth, proliferation, metastasis, and vascular growth of ESCC by secreting several growth factors, chemokines, and matrix metalloproteinases.^8^ However, the effects of CAFs on lymphangiogenesis in ESCC TME have rarely been reported.

Exosomes, approximately 40–150 nm in diameter, have a lipid bilayer membrane and are secreted by almost every type of cell.^9^ Johnstone isolated vesicle‐like structures from the medium of cultured reticulocytes and named them exosomes, although at that time, researchers believed that these particles were simply a type of cellular waste.^10^ However, we now know that exosomes contain many different components and transmit information across cells, thus contributing to various physiological and pathological events, including tumor development and metastasis.^11^, ^12^ Interestingly, exosomes are particularly abundant in the TME, indicating the active orchestration of different cell types in TME to promote cancer progression.

MicroRNAs (miRNAs) are short non‐coding RNAs made up of 21–24 nucleotides. By binding to mRNA 3′‐UTRs, miRNAs regulate target genes, thus modulating post‐transcriptional regulation of specific genes.^13^, ^14^ As one of the main constituents of exosomes, miRNAs function in a paracrine manner to play their biological roles in tumor initiation, development, and recurrence.^15^ Although a great amount of studies demonstrated different impact of exosomal miRNAs on ESCC tumor cells, the roles of exosome‐derived miRNAs in lymphangiogenesis in the ESCC TME are largely understudied. Several tumors have been associated with miR‐100‐5p, a member of the microRNA‐99 family. miR‐100‐5p is also involved in tumor proliferation and metastasis.^16^, ^17^ Several studies revealed decreased miR‐100‐5p expression in ESCC, and this decrease was linked to poor outcomes.^18^, ^19^ However, the exosome‐mediated effect of miR‐100‐5p on lymphangiogenesis in ESCC is unknown.

In this study, we found that CAFs in ESCC correlate with microlymphatic vessel density (MLVD), and CAF‐derived exosomes promote lymphangiogenesis in vitro and in vivo. Further mechanistic investigations suggested that decrease of exosomal miR‐100‐5p levels in CAFs leads to activation of IGF1R/PI3K/AKT pathway in endothelial cells, thus accelerating lymphangiogenesis. Our work highlights the key role of CAFs in lymph metastasis of ESCC and prospective molecular target for future therapy.

## 2 MATERIALS AND METHODS

### 2.1 Clinical samples

Surgically removed ESCC tissues were collected for examination. 6 fresh and 53 paraffin‐embedded samples of ESCC tissue without necrosis were selected. For control experiments, six fresh, and 25 paraffin‐embedded samples of normal esophageal tissue (>5 cm from cancer tissue) were selected. The patient selection criteria for ESCC were as follows: (1) Pathological diagnosis of ESCC, (2) No pre‐surgical treatment with radiotherapy, chemotherapy, or any other therapy, and (3) Availability of complete and reliable medical records. During the study, all patients provided written informed consent for tissue collection. The Zhengzhou University Bioethics Committee approved the study.

### 2.2 Hematoxylin–eosin (HE) staining and immunohistochemistry

Tissue was fixed in 4% paraformaldehyde for 12 h and then paraffin‐embedded before sectioning (4 μm). For HE staining, the sections were stained with hematoxylin and eosin dyes (Servicebio), washed with water, and then mounted using neutral gum after dehydration. For immunohistochemistry, antigen retrieval was performed using an EDTA (pH = 9.0) solution. Subsequently, endogenous peroxidase was inactivated using 3% hydrogen peroxide. The sections were incubated with primary antibodies for overnight at 4°C after blocked in normal goat serum (15 min at 25°C). Antibodies used were: α‐SMA (Abcam), FAP (Abcam), LYVE‐1 (Abcam), and D2‐40 (Zsbio). The diluent used was a special antibody diluent. After washing, sections were incubated with a biotin‐tagged secondary antibody (Zsbio) and then with a streptavidin‐HRP antibody (Zsbio) (each incubation, 15 min at 25°C). Then, DAB staining (Zsbio) was performed followed by hematoxylin staining. The sections were washed, dried, and mounted. Sections were imaged using an IX73 system (Olympus, Japan), and three random high‐power lens fields were selected to measure the number of CAFs and the MLVD.

### 2.3 Cell culture

EC9706 is a primary ESCC cell line obtained from the Cancer Institute's National Key Laboratory of Molecular Oncology. Human lymphocyte endothelial cells (HLECs) were purchased from Bena Culture Collection. EC9706 cells and HLECs were cultured in 100‐mm dishes containing DMEM, 10% fetal bovine serum (FBS), and 1% penicillin–streptomycin (37°C in 5% CO_2_). Cells were subcultured in the logarithmic growth stage, and some cells were preserved in liquid nitrogen for subsequent experiments.

### 2.4 Fibroblast isolation

The ESCC or normal esophageal tissues was stored in DMEM/F12 with 10% FBS and 1% penicillin–streptomycin (4°C). Repeated washes with Hank's balanced salt solution containing 10% penicillin–streptomycin were performed to remove blood. Tissue was cut (<1 mm^3^ pieces) and digested in DMEM/F12 complete medium supplemented with 0.1% type I collagenase (3 h, 25°C, with shaking). The mixture was centrifuged at 1000 rpm for 5 min, and the pellet was plated in 100‐mm dishes containing DMEM/F12 complete medium. When cells covered 80%–90% of the whole dish, they were passaged. Cells were plated in a 100‐mm dish and placed in an incubator for 20 min at 37°C. Then, the dish was washed to remove non‐adherent cells, and DMEM/F12 complete medium was added for culturing. These steps were repeated until cells with a more consistent shape were obtained.

### 2.5 Western blotting

BCA Protein Assay Kit was used to measure the total protein concentration in cells and tissue lysed in RIPA buffer. Then, 20 μg of proteins were separated using SDS‐PAGE at 80 V for 1 h, and the separated proteins were transferred to PVDF. After blocking with 5% skim milk (2 h, 25°C), primary antibodies were incubated overnight at 4°C. The antibodies used were: GAPDH (Abcam), α‐SMA (Abcam), FAP (Abcam), CD63 (Abcam), D81 (Abcam), calnexin (Abcam), IGF1R (CST), AKT (CST), p‐AKT (CST), PI3K (Wanleibio). The diluent used was a special western blotting antibody dilution buffer. The PVDF membranes were then washed and incubated with a secondary antibody conjugated with HRP (Cwbio) (1 h, 25°C). ECL substrate (Invitrogen) was used to obtain protein bands.

### 2.6 Immunofluorescence staining

Two sterile circular cover glasses (φ14 mm) (NEST, China) were placed in each well of a 6‐well plate (Corning). Then, 1 × 10^5^ cells were added to each well (incubation, 12 h). The cells were fixed with 4% paraformaldehyde (Saint‐bio) (30 min, 25°C) after they became tightly adherent. Then, they were incubated with 0.1% TritonX‐100 solution (Saint‐bio) for 20 min. The cells were blocked with normal goat serum (Cwbio) (30 min, 25°C), then incubated overnight at 4°C with primary antibodies. Antibodies used were as follows: α‐SMA (Abcam) and FAP (Bioss). The diluent used was a special antibody diluent (Cwbioa). After washing, cells were treated with goat anti‐rabbit IgG H&L Alexa Fluor® 488 (Abcam) (1 h, 25°C) in the dark. They were then stained with DAPI (Abcam) for 10 min at the same conditions. The IX73 system (Olympus) was used to view cells and capture images.

### 2.7 Exosome isolation

After culturing cells in DMEM/F12 without FBS (48 h), medium was filtered with a 0.22 μm filter (Millipore) and centrifuged (3000 × g, 15 min). The supernatant was transferred into a sterile centrifuge tube and concentrated through an ultrafiltration centrifugation tube (Millipore). The concentrate was added to an ExoQuick‐TC exosome precipitation solution (SBI) at a ratio of 5:1 (vol/vol). The mixture was incubated overnight at 4°C. Subsequently, centrifugation was performed (1500 × g, 30 min, 4°C). The precipitate (exosomes) was collected and resuspended in PBS (Hyclone). Exosomes were preserved at −80°C for later experiments.

### 2.8 Transmission electron microscopy

Exosomes were placed on the carbon‐coated copper mesh (5 min), and 2% phosphotungstic acid was added. After 3 min, the carbon‐coated copper mesh was dried at 25°C. Images of exosomes were collected using an HT7800 system (Hitachi) at 80 kV.

### 2.9 NanoSight particle tracking analysis

Exosomes, diluted with PBS, were slowly injected into the sample unit of ZetaView PMX 110 (PMX) and detected. The concentration and size of the exosomes were examined using ZetaView 8.04.02 analysis software.

### 2.10 Induction of HLECs in conditioned medium

The supernatant of EC9706 cells was collected, centrifuged, and filtered to obtain conditioned medium for culturing HLECs. HLECs were induced into tumor‐associated lymphatic endothelial cells (TLECs) after 48 h of culture. Then, the function of TLECs was verified. TLECs were subcultured in the logarithmic growth stage for subsequent experiments.

### 2.11 Exosome labeling and tracking

The lipid bilayer of exosomes was labeled using a PKH26 Red Fluorescent Cell Linker Mini Kit (Sigma). First, 1 × 10^5^ cells were plated in a 35‐mm glass bottom dish (NEST) and incubated for 12 h. Then, labeled exosomes were added to DMEM without FBS and incubated for 24 h. After washing, cells were incubated with DAPI (10 min, 25°C, in the dark). Images were acquired using an LSM800 system (ZEISS).

### 2.12 Cell migration assay

First, 2 × 10^5^ cells were plated in a 6‐well plate and incubated for 24 h. A scratch was introduced in the center of 6‐well plate using a 100–1000 μL pipette tip (Axygen). The plate was washed to remove floating cells, and exosomes were added (2 μg exosomes in DMEM based on protein levels, added to 1 × 10^5^ cells). Images of the scratch were collected at 0 and 24 h after processing. ImageJ was used to detect and analyze the area of the scratch.

### 2.13 Cell invasion assay

First, 50 μL extracellular matrix gel was added to the transwell chamber (Falcon) and placed in an incubator for 30 min. Then, 5 × 10^4^ cells were added and cultured with DMEM containing exosomes for 24 h. Crystal violet was stained on the lower surface of the membrane (30 min, 25°C) after the upper surface of the membrane was carefully erased. Images were obtained using a microscope, and three random fields were chosen for cell counting.

### 2.14 Cell proliferation assay

First, 5 × 10^3^ cells were plated in a 96‐well plate (Corning) and cultured in DMEM containing exosomes. Then, 10 μL Cell Counting Kit‐8 reagent (Dojindo) was added (incubation, 2 h). Optical density (OD) at 450 nm was detected after 24, 48, 72, and 96 h.

### 2.15 Cell tube formation assay

First, 250 μL extracellular matrix gel was added to a 24‐well plate (Corning) (incubation, 30 min). Then, 1 × 10^5^ cells were added to each well and cultured with DMEM containing exosomes. Images were collected 6 h after processing. ImageJ software was used for detecting and analyzing tube formation.

### 2.16 RNA extraction and qRT‐PCR

TRIzol reagent (Cwbio) was used to obtain total RNA from tissues or cells. The miRNA first‐strand cDNA synthesis kit (Vazyme) was used to reverse transcribe miRNA. The miRNA Universal SYBR qPCR Master Mix (Vazyme) and QuantStudio5 real‐time PCR system (Thermo) were used for the real‐time quantitative PCR. The primers (10 μM) were synthesized by Wuhan Servicebio Technology Co., Ltd. 2^−ΔΔCq^ method was used to calculate miRNA relative expression levels. The primer sequences (5′‐3′) are shown in Table 1.

**TABLE 1:** Primer sequences.

PCR primer	Sequence (5′‐3′)
U6	F‐CTCGCTTCGGCAGCACA
	R‐AACGCTTCACGAATTTGCGT
let‐7c‐5p	RT‐CTCAACTGGTGTCGTGGAGTCGGCAATTCAGTTGAGAACCATAC
	F‐ACACTCCAGCTGGGTGAGGTAGTAGGTTGT
	R‐TGGTGTCGTGGAGTCG
miR‐31‐5p	RT‐CTCAACTGGTGTCGTGGAGTCGGCAATTCAGTTGAGAGCTATGC
	F‐ACACTCCAGCTGGGAGGCAAGATGCTGGC
	R‐TGGTGTCGTGGAGTCG
miR‐100‐5p	RT‐CTCAACTGGTGTCGTGGAGTCGGCAATTCAGTTGAGCACAAGTT
	F‐ACACTCCAGCTGGGAACCCGTAGATCCGAA
	R‐TGGTGTCGTGGAGTCG
miR‐142‐5p	RT‐CTCAACTGGTGTCGTGGAGTCGGCAATTCAGTTGAGAGTAGTGC
	F‐ACACTCCAGCTGGGCATAAAGTAGAAAGC
	R‐TGGTGTCGTGGAGTCG

Abbreviations: F, forward; R, reverse; RT, reverse transcription.

### 2.17 Transfection

First, 2 × 10^5^ cells were plated in a 6‐well plate and incubated for 24 h. Cells were separately transfected with an LV3‐NC, miR‐100‐5p mimic, or miR‐100‐5p inhibitor lentivirus (GenePharma) for 48 h. Cells showing stable expression were selected after 72 h of incubation with DMEM complete medium containing puromycin (2 μg/mL) (Sigma).

### 2.18 Dual luciferase reporter assay

First, 2 × 10^5^ cells were plated in a 6‐well plate and incubated for 24 h. The dual luciferase reporter plasmids, wild‐type/mutant IGF1R and miR‐100‐5p mimic/LV3‐NC (GenePharma), were co‐transfected using Lipofectamine 2000 (GenePharma) (duration, 48 h). Luciferase activity was detected using a Dual Luciferase Assay Kit (Vazyme).

### 2.19 Fluorescence in situ hybridization

The probes of miR‐100‐5p and IGF1R were designed and synthesized by GenePharma Co., Ltd. Then, Fluorescence in situ hybridization kit (GenePharma) was used to detect the expression and cellular localization of miR‐100‐5p and IGF1R according to the instructions. Images were acquired using an LSM800 system (ZEISS).

### 2.20 Mouse tumor models and treatment

25 BALB/c nude female mice were obtained from Beijing Vital River Laboratory Animal Technology Co., Ltd. Then, 5 × 10^6^ EC9706 cells resuspended in 200 μL PBS were injected subcutaneously in their right flanks. After 1 week, the mice were divided into the following groups and the following exosomes/constructs were injected into the tumors: NF‐derived exosomes group, 50 μL NF‐derived exosomes (5 μg exosomes in DMEM based on protein levels); CAF‐derived exosomes group, 50 μL CAF‐derived exosomes (5 μg exosomes in DMEM based on protein levels); LV3‐NC group, 50 μL LV3‐NC lentivirus (1 × 10^9^ TU/mL); miR‐100‐5p mimic group: 50 μL miR‐100‐5p mimic lentivirus (1 × 10^9^ TU/mL); and miR‐100‐5p inhibitor group, 50 μL miR‐100‐5p inhibitor lentivirus (1 × 10^9^ TU/mL). All injections were carried out every 4 days, and a total of four injections were administered. After 2 weeks, all mice were sacrificed, and their tumors were removed, measured, and weighed. The Experimental Animal Ethics Committee of Henan Institute of Medical and Pharmaceutical Sciences approved all animal experiments, which followed Chinese animal care and institutional ethics.

### 2.21 Statistical analysis

All data were analyzed using GraphPadPrism8.0 (GraphPad) and represented as means ± standard deviations. All experiments were repeated thrice. The difference between two groups was analyzed using the *t*‐test, and the difference between several groups was analyzed using the one‐way analysis of variance (ANOVA). Pearson's method was used for correlation analyses. Differences were considered statistically significant at *p* < 0.05.

## 3 RESULTS

### 3.1 CAFs are involved in ESCC lymphangiogenesis

**FIGURE 1:**
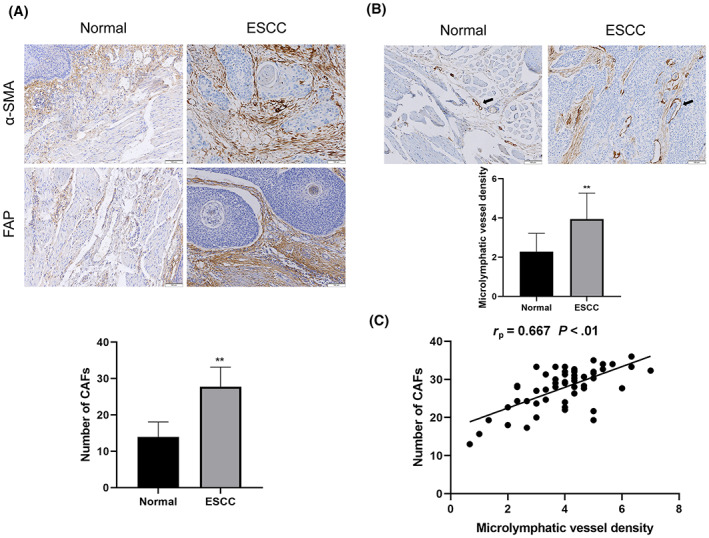
CAFs are associated with lymphangiogenesis in ESCC. (A) Immunohistochemical staining showing CAFs labeled with α‐SMA and FAP. The number of CAFs in the normal esophageal tissue was significantly lower than that in ESCC (100×). (B) Immunohistochemical staining showing lymphatic endothelial cells labeled with D2‐40. The MLVD in the normal esophageal tissue was significantly lower than that in ESCC (100×). (C) Correlation between the number of CAFs and MLVD in ESCC. *p < 0.05, **p < 0.01.

To examine the relationship between CAFs and lymphangiogenesis in ESCC, we performed immunohistochemistry analysis in a cohort of human ESCC samples. α‐SMA and FAP, two independent specific markers of CAFs, expressed in cytoplasm and cell membrane was investigated, respectively. A large number of α‐SMA‐ and FAP‐positive CAFs (Figure 1A) were present in ESCC samples, the number of which was higher in these samples than in normal esophageal tissue. Meanwhile, D2‐40, a specific marker of lymphatic endothelial cells, was examined in the same cohort. The positive signals of D2‐40 immunostaining are typical biomarker indicating MLVD. We found that a large number of nascent microlymphatic vessels were observed in ESCC, and the MLVD in the normal esophageal tissue was significantly lower (Figure 1B).

The correlations between CAFs/MLVD and the clinicopathological features of ESCC patients —including gender, age, tumor diameter, depth of invasion, lymphatic metastasis, and pathological stage—were analyzed. The results showed that the number of CAFs and MLVD were not associated with gender or age. However, these factors were associated with pathological stage, depth of infiltration, and the presence of lymphatic metastasis (Tables 2, 3). Importantly, a strong positive correlation between the number of CAFs and MLVD in ESCC tissues was identified (Figure 1C). Together, the results indicated that CAFs were associated with ESCC lymphangiogenesis, thus affecting the progression of ESCC.

**TABLE 2:** Correlation between the number of CAFs in ESCC tissues and clinicopathological features in ESCC patients.

Variable	Cases (*N*)	Number of CAFs	*t*	*p*
Gender				
Male	35	27.88 ± 5.45	0.251	0.803
Female	18	27.46 ± 6.11		
Age (years)				
≤60	14	29.67 ± 3.91	1.515	0.136
>60	39	27.04 ± 6.02		
Histological grade				
G1	17	25.39 ± 6.68	2.155	0.036*
G2‐G3	36	28.84 ± 4.77		
Lymphatic metastasis				
No	28	25.25 ± 5.73	3.821	0.000**
Yes	25	30.52 ± 4.21		
Depth of invasion				
Fibrous membrane	32	26.27 ± 5.65	2.449	0.018*
Superficial and deep muscularis	21	29.97 ± 4.93		

* *p* < 0.05.

** *p* < 0.01.

**TABLE 3:** Correlation between MLVD in ESCC tissues and clinicopathological features in ESCC patients.

Variable	Cases (*N*)	MLVD	*t*	*p*
Gender				
Male	35	4.08 ± 1.33	1.068	0.291
Female	18	3.67 ± 1.30		
Age (years)				
≤60	14	4.21 ± 1.21	0.912	0.366
>60	39	3.84 ± 1.36		
Histological grade				
G1	17	3.33 ± 1.48	2.382	0.021*
G2‐G3	36	4.22 ± 1.16		
Lymphatic metastasis				
No	28	3.40 ± 1.15	3.398	0.001**
Yes	25	4.53 ± 1.28		
Depth of invasion				
Fibrous membrane	32	3.57 ± 1.38	2.606	0.012*
Superficial and deep muscularis	21	4.49 ± 1.03		

* *p* < 0.05.

** *p* < 0.01.

### 3.2 Identification of CAFs and CAF‐derived exosomes

**FIGURE 2:**
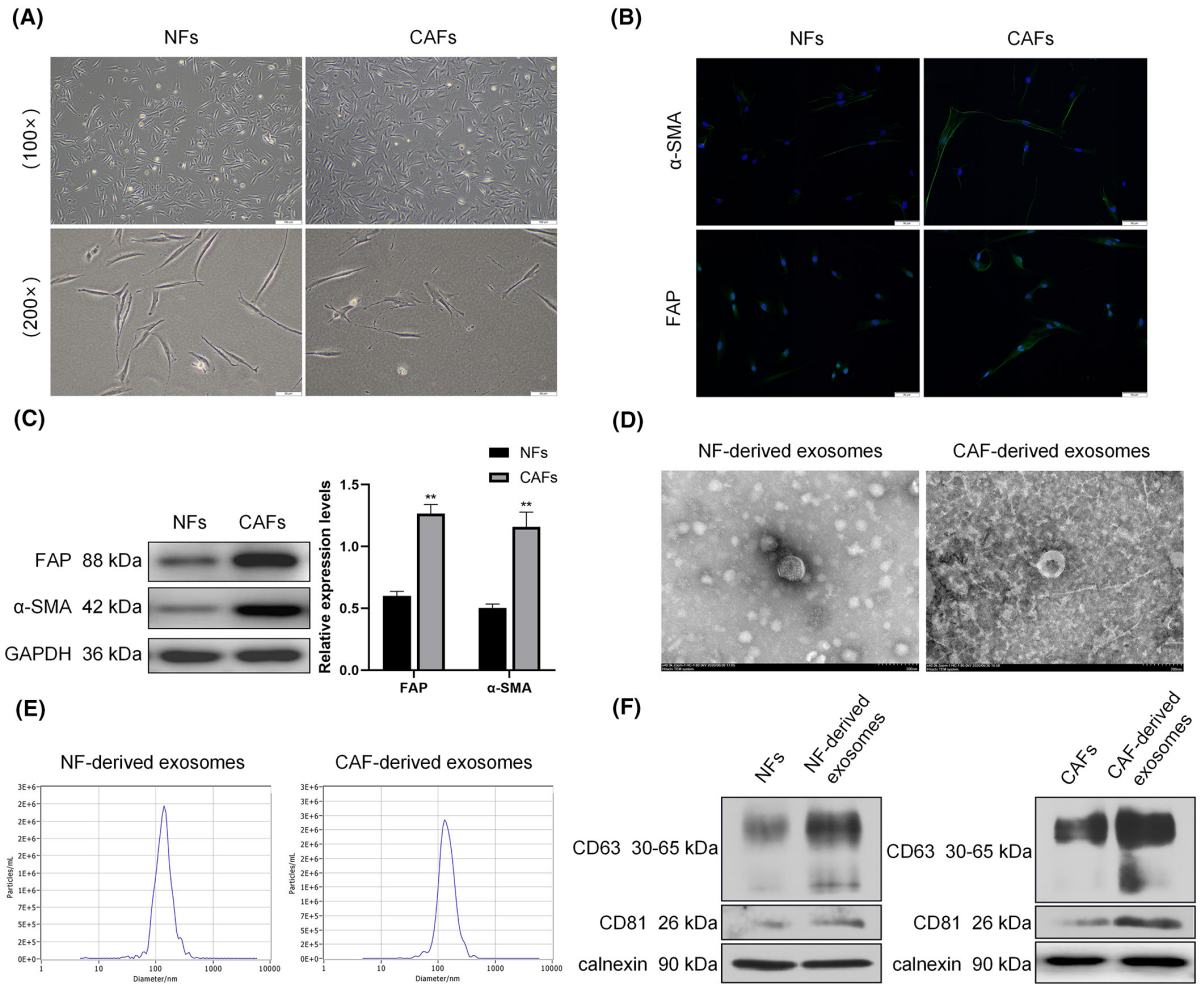
Identification of CAFs and CAF‐derived exosomes. (A) Image showing the primary CAFs and NFs. (B) Immunofluorescence staining and (C) western blotting showing α‐SMA and FAP expression in CAFs and NFs. (D) Transmission electron microscopy images showing exosomes derived from CAFs and NFs. (E) NanoSight particle tracking analysis showing the size and concentration distribution of exosomes. (F) Western blotting showing CD63, CD81, and calnexin expression in exosomes and cells. *p < 0.05, **p < 0.01.

Human primary CAFs and normal fibroblasts (NFs) were obtained using the differential adherence method. Microscopic examinations showed that CAFs and NFs were mostly long spindle‐shaped, although some cells were triangular or polygonal in shape. However, CAFs had more protrusions (Figure 2A). Immunofluorescence staining (Figure 2B) showed that the fluorescence intensity for both α‐SMA and FAP was higher in CAFs than in NFs. These findings were confirmed by immunoblotting analysis (Figure 2C), which revealed higher expression levels of the two proteins in CAFs relative to NFs. These findings confirmed the successful isolation of CAFs and NFs.

Transmission electron microscopy showed that the exosomes were vesiculate and approximately 100 nm in diameter (Figure 2D). By ZetaView PMX analysis, more than 97% of the isolated exosomes were approximately 140 nm in diameter (Figure 2E). The expression of CD63 and CD81, which are specific protein markers of exosomes, and calnexin, which is an ER membrane marker, were examined (Figure 2F). The results showed that both CD63 and CD81 levels were higher in exosomes than in cells, but calnexin was not expressed in exosomes. This confirmed the successful isolation of exosomes from CAFs and NFs.

### 3.3 CAF‐derived exosomes promote lymphangiogenesis in vitro

**FIGURE 3:**
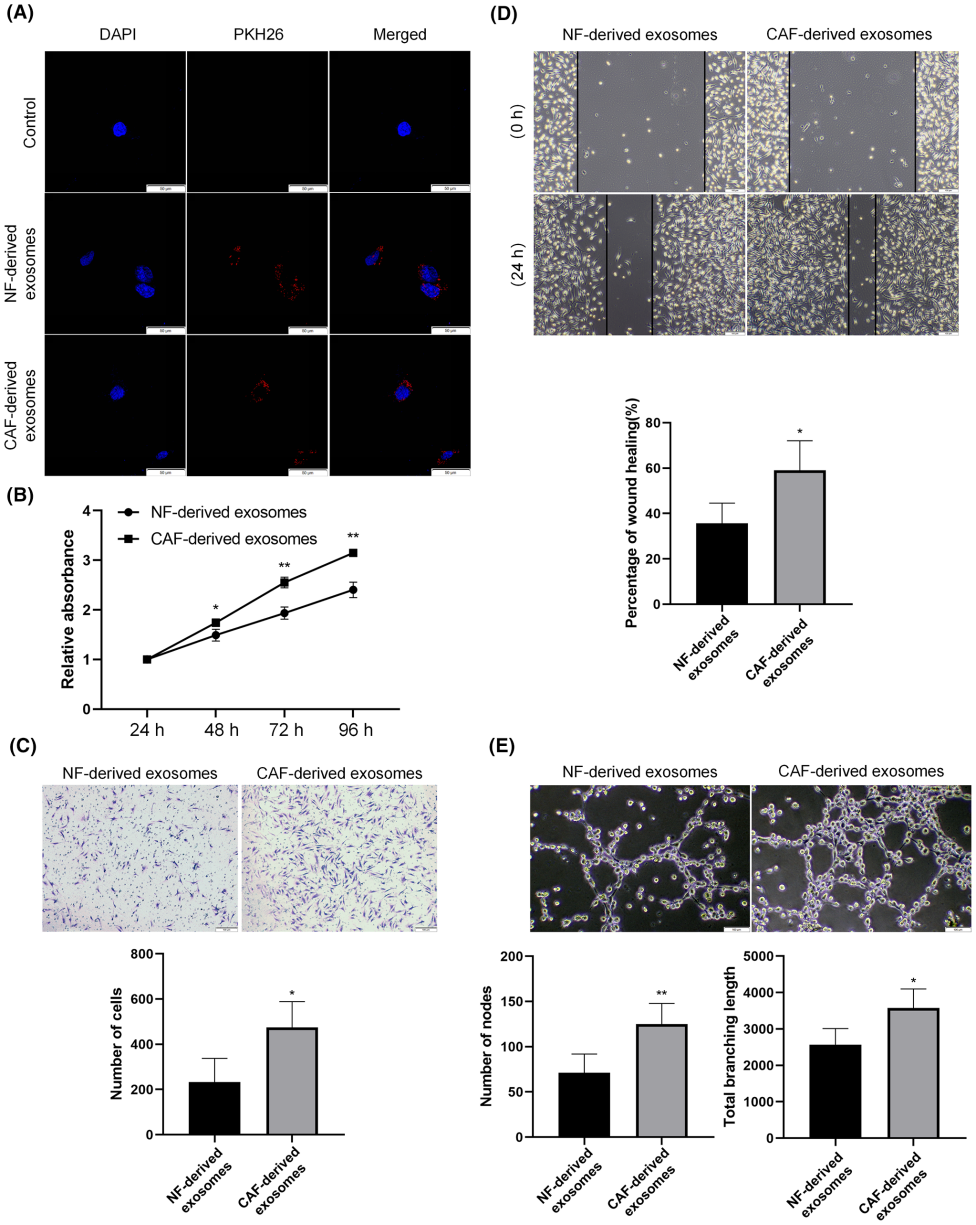
CAF‐derived exosomes promote lymphangiogenesis in vitro. (A) Exosomes labeled with the PKH26 dye entered TLECs and accumulated in the nucleus. (B) CCK‐8 assay showing the proliferation of TLECs cultured with exosomes. (C) Cell invasion assay showing the invasion ability of TLECs cultured with exosomes. (D) Scratch experiment showing the migration capacity of TLECs cultured with exosomes. (E) Cell tube formation assay showing the tube formation capacity of TLECs cultured with exosomes. *p < 0.05, **p < 0.01.

In order to simulate the TME of ESCC, HLECs were induced into TLECs using conditioned medium containing supernatant from EC9706 cells. TLECs showed the better cell proliferation, migration, invasion, and tube formation ability than HLECs (Figure S1). To further examine whether exosomes secreted by CAFs display any impact on polarized TLECs, we first sought to test the ability of CAF‐derived exosomes to enter TLECs. PKH‐26 is a lipophilic dye, which can stably bind to the lipid region of exosomes and emit fluorescence, and is a commonly used in exosome research. TLECs were cultured with exosomes labeled using the PKH26 dye, and fluorescent microscopy showed that exosomes were able to enter TLECs and became concentrated inside their nuclei (Figure 3A). Next, we performed several functional studies in a system of TLECs co‐cultured with CAF‐derived exosomes. In the presence of the exosomes, TLECs exhibited a higher rate of proliferation and increased number of TLECs crossing the transwell chamber (Figure 3B,C). Likewise, TLECs co‐cultured with CAF‐derived exosomes showed a better scratch healing ability, that is, better migration capacity (Figure 3D), as well as increased number of nodes and a longer total branching length, indicating a higher tube formation capacity (Figure 3E). These results indicated that CAF‐derived exosomes promote lymphangiogenesis in vitro.

### 3.4 CAF‐derived exosomes promote lymphangiogenesis in a xenograft mouse model of ESCC

**FIGURE 4:**
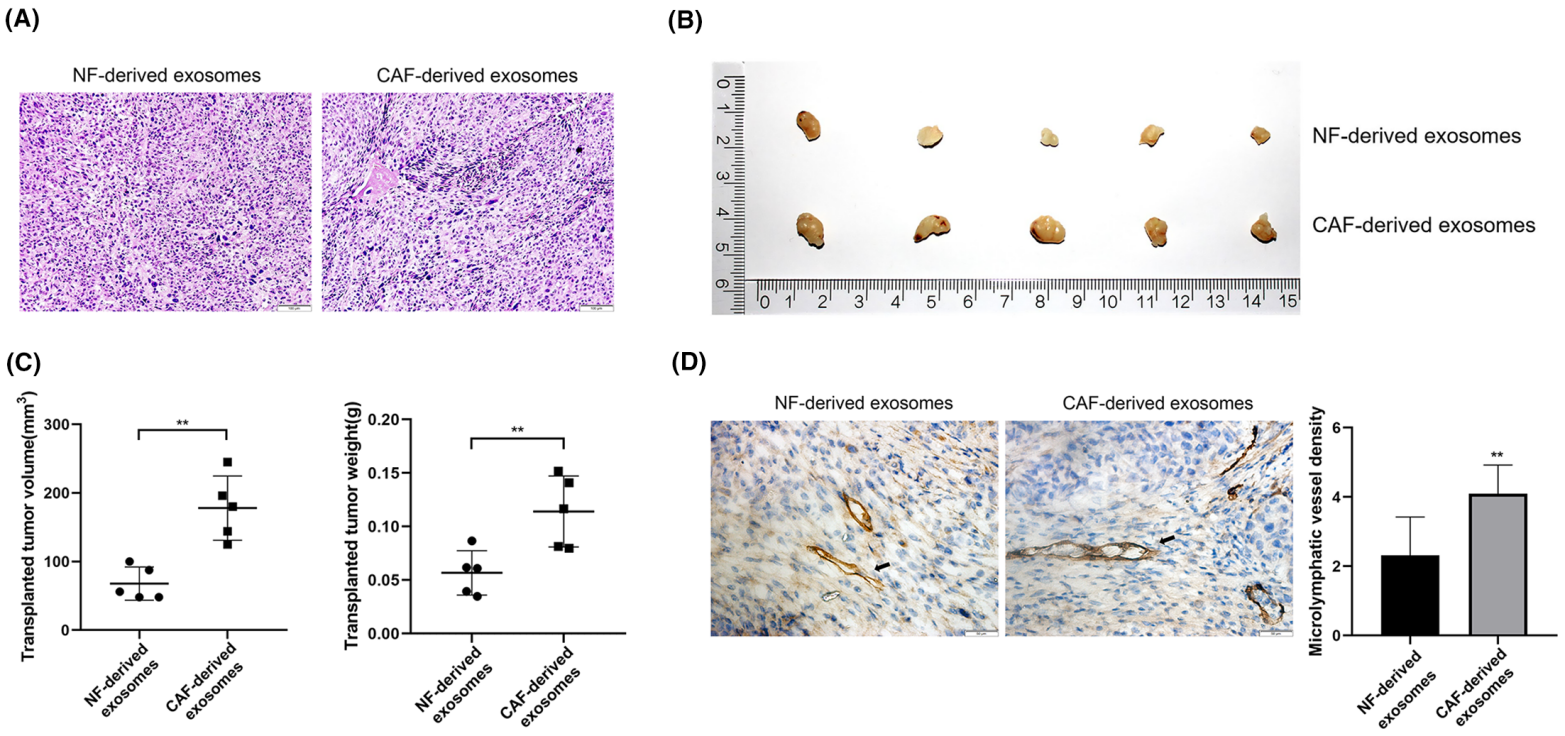
CAF‐derived exosomes promote lymphangiogenesis in a xenograft mouse model of ESCC. (A) Image showing tumors from a xenograft mouse model of ESCC treated with different types of exosomes. (B) Weight and volume of tumors in the NF‐derived exosomes and CAF‐derived exosomes groups. (C) HE staining showing the structure and cell morphology of tumors (100×). (D) Immunohistochemical staining showing lymphatic endothelial cells labeled with LYVE‐1 (200×). The MLVD in tumors from the CAF‐derived exosomes group was significantly higher than that in tumors from the NF‐derived exosomes group. *p < 0.05, **p < 0.01.

To validate the findings in the cell co‐culture system, we thus examined the effects of CAF‐derived exosomes on subcutaneous ESCC xenografts. HE staining was used for the pathological examination of the tumors (Figure 4A). The tumors showed the typical structure and cell morphology of squamous carcinoma. Further, several cells showed abnormal nuclear division, indicating the successful establishment of the ESCC xenograft mouse model. Compared to NF‐derived exosomes treatment, the tumor weight, and volume were significantly higher in the CAF‐derived exosomes group (Figure 4B,C), indicating the tumor promoting activity of CAF‐derived exosomes.

To visualize lymphatic endothelial cells in the tumors, we labeled the cells with antibody against LYVE‐1, a protein predominantly expressed in lymphatic endothelial cells displaying a punctate distribution on the surface of the lymphatic lumen and basolateral plasma membrane (Figure 4D). The tumor MLVD was further determined by LYVE‐1 positive signals. Consistent with the result in human ESCC samples, the MLVD in the CAF‐derived exosomes group was higher than that in the NF‐derived exosomes group (Figure 4D). This demonstrated that CAF‐derived exosomes also promote lymphangiogenesis in a ESCC xenograft mouse model.

### 3.5 miR‐100‐5p inhibits lymphangiogenesis in vitro

**FIGURE 5:**
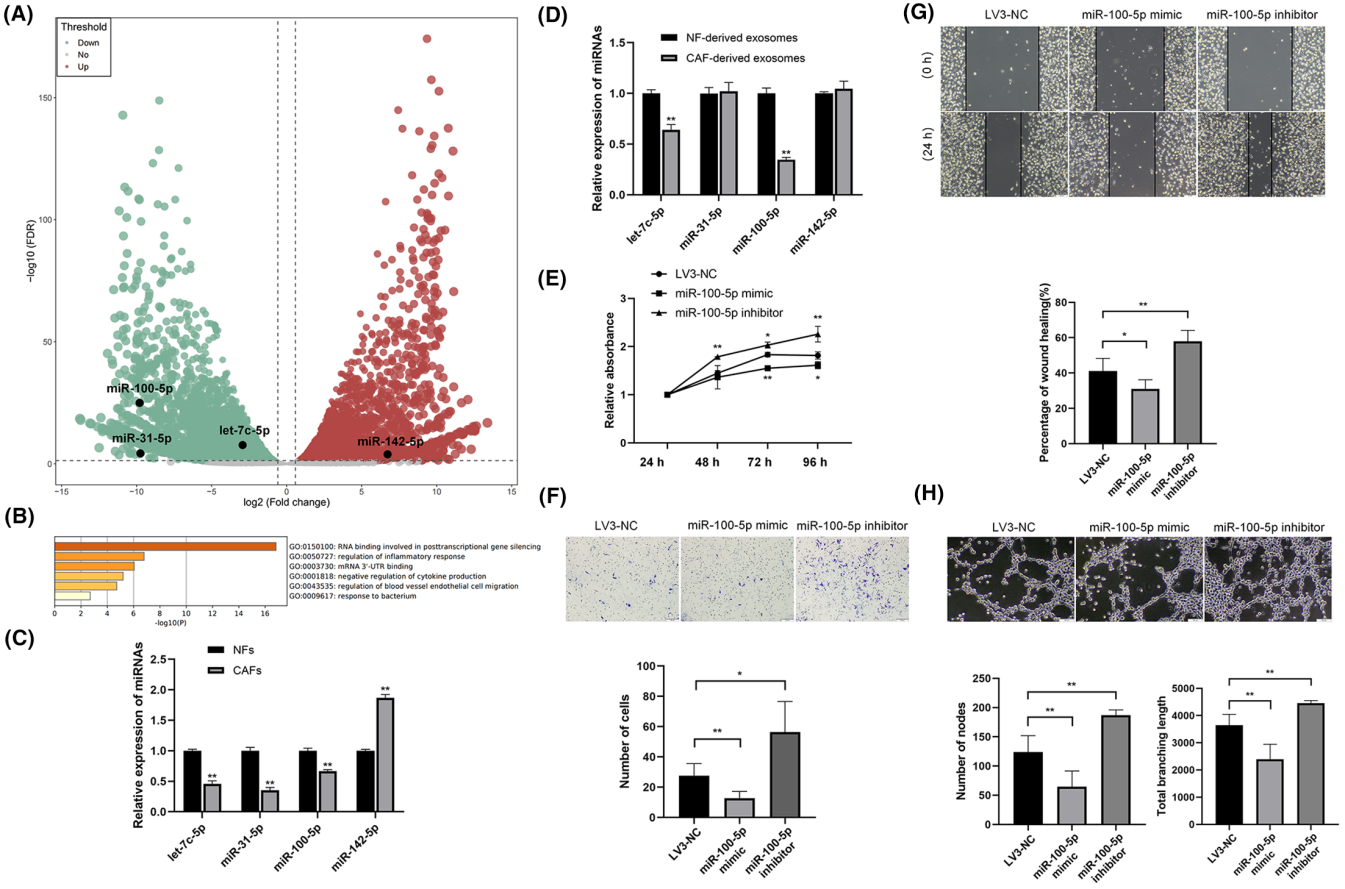
miR‐100‐5p inhibits lymphangiogenesis in vitro. (A) The volcano plot for dataset GSE103111. (B) Functional annotation analysis of the differentially expressed miRNAs. qRT‐PCR showing the levels of candidate miRNAs in (C) cells and (D) exosomes. (E) CCK‐8 assay showing the proliferation of TLECs, (F) cell invasion assay showing the invasion ability of TLECs, (G) scratch assay showing the migration capacity of TLECs, (H) and cell tube formation assay showing the tube formation capacity of TLECs in the LV3‐NC, miR‐100‐5p mimic, and miR‐100‐5p inhibitor groups. *p < 0.05, **p < 0.01.

The dataset GSE103111 was selected from the GEO database (https://www.ncbi.nlm.nih.gov/geo/). It contained high‐throughput sequencing data for CAFs and matched NFs obtained from nine ESCC patients. The data were analyzed using the Limma software package of R to identify differentially expressed RNAs and draw the volcano plot (Figure 5A). In total, 22 miRNAs were identified. These 22 miRNAs were subjected to functional enrichment analysis using Metascape (https://metascape.org/gp/index.html#/main/step1). Among these, 14 miRNAs could be functionally classified according to GO terms (Figure 5B). Based on expression differences and the function of the miRNAs, let‐7C‐5p, miR‐31‐5p, miR‐100‐5p, and miR‐142‐5p were selected as candidate miRNAs for screening. The levels of the candidate miRNAs were examined in NFs, CAFs, NF‐derived exosomes, and CAF‐derived exosomes using qRT‐PCR (Figure 5C,D). miR‐100‐5p levels were found to be significantly lower in CAF‐derived exosomes than in NF‐derived exosomes, and the downregulation multiple was 0.35. Therefore, miR‐100‐5p was selected as the target miRNA for subsequent experiments.

Lentiviral constructs were designed and synthesized and then transfected into TLECs (Figure S2). Cell proliferation was found to be slowest in miR‐100‐5p mimic‐transfected TLECs (Figure 5E). Invasion assays revealed that the number of TLECs crossing the transwell chamber was lowest in the miR‐100‐5p mimic group (Figure 5F). The scratch assay (Figure 5G) and tube formation assay (Figure 5H) revealed that the scratch healing speed and tube formation capacity were lowest in TLECs from the miR‐100‐5p mimic group. The findings suggested that miR‐100‐5p upregulation inhibits lymphangiogenesis in vitro.

### 3.6 miR‐100‐5p inhibits lymphangiogenesis in a xenograft mouse model of ESCC

**FIGURE 6:**
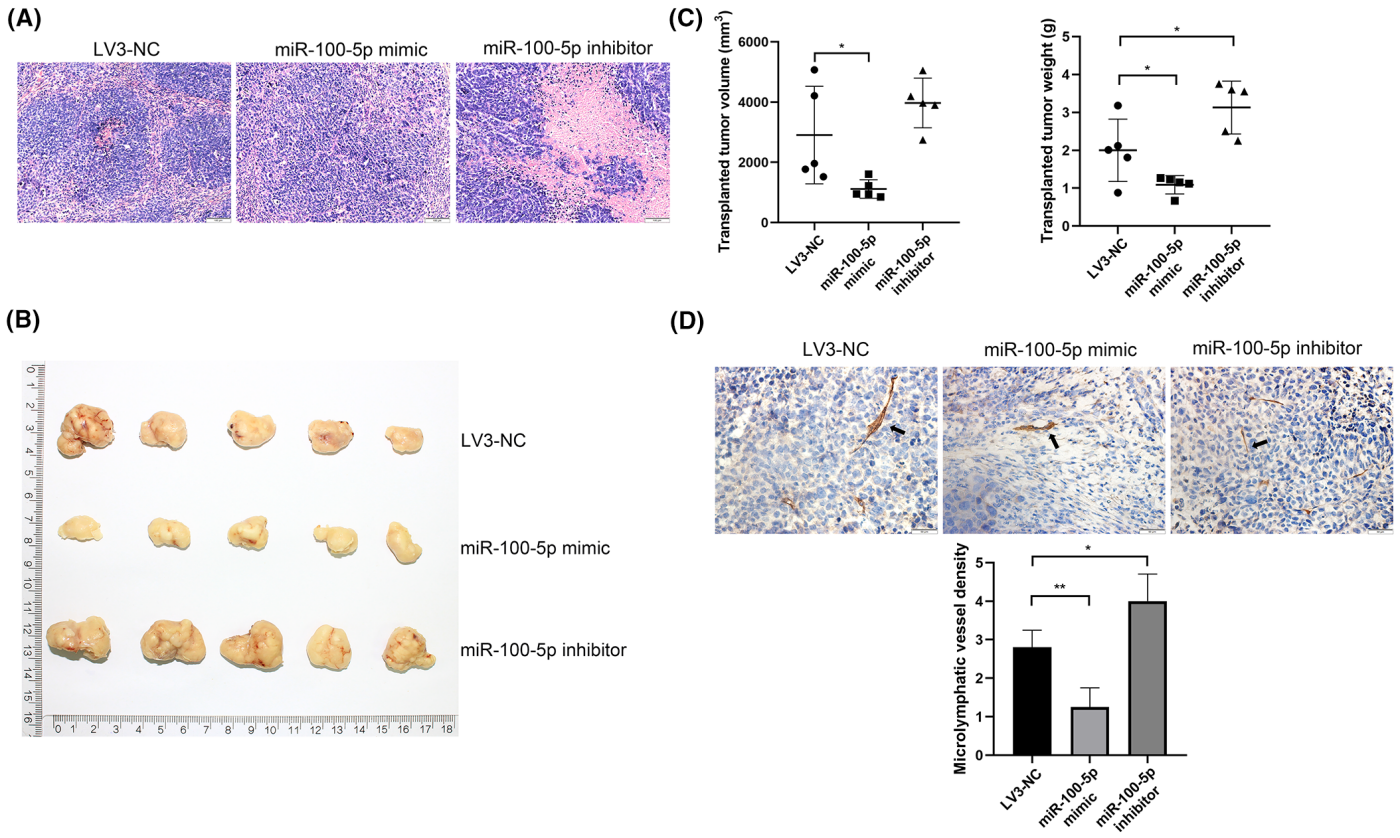
miR‐100‐5p inhibits lymphangiogenesis in a xenograft mouse model of ESCC. (A) Image showing tumors from a xenograft mouse model of ESCC treated with different lentiviral constructs. (B) Weight and volume of tumors in the LV3‐NC, miR‐100‐5p mimic, and miR‐100‐5p inhibitor groups. (C) HE staining showing the structure and cell morphology of tumors (100×). (D) Immunohistochemical staining showing lymphatic endothelial cells labeled with LYVE‐1 (200×). The MLVD in tumors from the LV3‐NC, miR‐100‐5p mimic, and miR‐100‐5p inhibitor groups. *p < 0.05, **p < 0.01.

HE staining was used for the pathological examination of the tumors treated with different lentiviral constructs (Figure 6A). The tumors showed the typical structure and cell morphology of squamous carcinoma, and several cells showed abnormal nuclear division. The tumors in the miR‐100‐5p inhibitor group showed large areas of necrosis, which could have resulted from rapid tumor growth and insufficient nutrient supply. After treatment with different lentiviral constructs (Figure 6B), tumor weight and volume were examined (Figure 6C). The miR‐100‐5p mimic group showed the lowest tumor weight and volume, followed by the LV3‐NC and miR‐100‐5p inhibitor groups. This indicated that miR‐100‐5p inhibits tumor growth.

LYVE‐1 was used to label these cells (Figure 6D), and the MLVD was determined. The MLVD was lowest in the miR‐100‐5p mimic group, followed by the LV3‐NC and miR‐100‐5p inhibitor groups. This demonstrated that miR‐100‐5p upregulation inhibits lymphangiogenesis in a xenograft mouse model of ESCC.

### 3.7 miR‐100‐5p inhibits lymphangiogenesis in ESCC via the IGF1R/PI3K/AKT pathway

**FIGURE 7:**
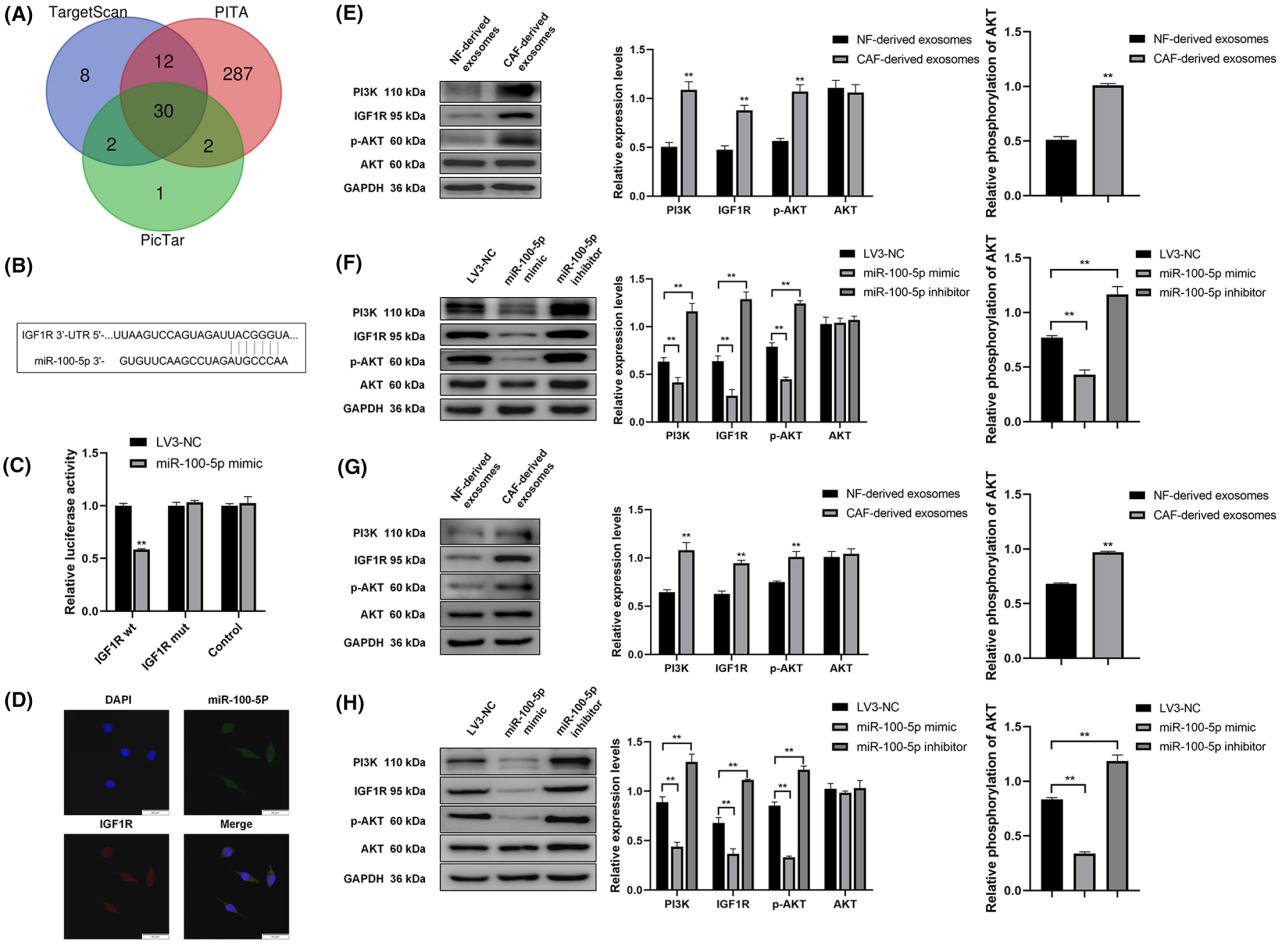
miR‐100‐5p inhibits lymphangiogenesis via the IGF1R/PI3K/AKT pathway. (A) Prediction of downstream targets of miR‐100‐5p using TargetScan, PITA, and PicTar. (B) Prediction of binding sites between miR‐100‐5p and IGF1R using a website tool. (C) Dual luciferase reporter assay showing the relationship of miR‐100‐5p and IGF1R. (D) Fluorescence in situ hybridization assay showing the co‐localization between miR‐100‐5p and IGF1R. green color: miR100‐5p, red color: IGF1R, blue color: DAPI. (E–H) Western blotting showing the expression of IGF1R, PI3K, AKT, and p‐AKT in (E) TLECs cultured with exosomes, (F) TLECs from the LV3‐NC, miR‐100‐5p mimic, and miR‐100‐5p inhibitor groups, (G) tumors injected with exosomes, and (H) tumors from the LV3‐NC, miR‐100‐5p mimic, and miR‐100‐5p inhibitor groups. *p < 0.05, **p < 0.01.

To predict downstream targets of miR‐100‐5p, TargetScan (http://www.targetscan.org/vert_71/), PITA (http://genie.weizmann.ac.il/pubs/mir07/mir07_data.html) and PicTar (https://pictar.mdc‐berlin.de/) were used (Figure 7A). Insulin‐like growth factor 1 receptor (IGF1R) was identified as a target based on the number of supported AGO CLIP‐seq experiments from ENCORI (https://starbase.sysu.edu.cn/). Dual luciferase reporter plasmids, which were designed according to the predicted binding sites of miR‐100‐5p and IGF1R (Figure 7B), were synthesized and transfected into TLECs. TLECs co‐transfected with miR‐100‐5p‐IGF1R wt reporter plasmids and miR‐100‐5p mimics showed a decrease in luciferase activity (Figure 7C). In addition, fluorescence in situ hybridization showed the co‐localization between miR‐100‐5p and IGF1R in cytoplasm of TLECs (Figure 7D). This suggested that IGF1R was a downstream target of miR‐100‐5p.

We explored whether IGF1R and the PI3K/AKT pathway, its classical downstream mediator that participates in cell growth, were involved in lymphangiogenesis in ESCC. IGF1R, PI3K, AKT, and p‐AKT expression was examined in cells and tumors using western blotting (Figure 7E–H). IGF1R, PI3K, and p‐AKT levels were found to be higher in TLECs cultured with CAF‐derived exosomes and tumors injected with CAF‐derived exosomes. Moreover, AKT phosphorylation levels were also higher in TLECs cultured with CAF‐derived exosomes and tumors injected with CAF‐derived exosomes. Additionally, IGF1R, PI3K, and p‐AKT levels were lowest in TLECs and tumors from the miR‐100‐5p mimic group, followed by the LV3‐NC and miR‐100‐5p inhibitor groups. AKT phosphorylation showed a similar trend. Hence, the findings implied that miR‐100‐5p targets IGF1R, thereby inhibiting PI3K/AKT pathway activation, and thus inhibits lymphangiogenesis in ESCC.

## 4 DISCUSSION

At present, standard therapy for esophageal cancer remains limited to surgery or endoscopic resection, radiotherapy, and chemotherapy. Although the 5‐year survival rate of ESCC increased from 20.9% in 2003 to 30.3% in 2012, it is still lower than that of most other cancers.^20^ Therefore, in order to improve patient outcomes, a better understanding of the molecular pathogenesis and metastasis of esophageal cancer is needed. There is increasing evidence that external factors such as the TME influence tumor development.^21^, ^22^ As an important component of the TME, CAFs are also closely involved in these processes. In previous co‐culture studies, researchers found that CAFs promote tumor progression to a greater degree than NFs.^23^ The effect of CAFs on tumor growth partially depend on their ability to induce angiogenesis.^24^ CAFs are known to promote lymph node metastases both in vivo and in vitro in ESCC, although the specific mechanisms have so far been unknown.^25^ Therefore, in this study, the number of CAFs in ESCC was detected using immunohistochemistry. Statistical analysis showed that the number of CAFs in ESCC was significantly higher than that in normal esophageal tissue, indicating that the fibroblasts in ESCC were typically activated. Then, we analyzed the correlation between the number of CAFs/MLVD and the clinicopathological features of ESCC, and found that these factors were associated with pathological stage, depth of infiltration, and the presence of lymphatic metastasis. Meanwhile, the MLVD was positively correlated with the number of CAFs. Therefore, our study indicated that CAFs affect ESCC lymphangiogenesis, thereby promoting ESCC progression.

Exosomes can carry and transport important signaling molecules, such as miRNA, LncRNA, and circRNA. Thus, the exosomal system represents a novel intercellular information transmission network and contributes to tumor occurrence and metastasis.^26^, ^27^, ^28^ Exosomes are particularly prevalent in the TME and mediate the effects of CAFs on the progression and metastasis of tumors. CAF‐derived exosomes can stimulate the growth and migration of ESCC cells.^29^ Several previous studies have described the effect of exosomes on lymphangiogenesis.^30^, ^31^, ^32^ However, whether CAF‐derived exosomes can promote lymphangiogenesis in ESCC has so far remained unclear. Therefore, in the present study, primary NFs and CAFs were isolated and used to extract exosomes. To simulate the TME of ESCC, we obtained TLECs induced using conditioned medium containing the supernatant of EC9706 cells. When exosomes were co‐cultured with TLECs, the cell proliferation, migration, invasion, and tube formation ability of TLECs treated with CAF‐derived exosomes improved. To further verify the role of CAF‐derived exosomes in ESCC lymphangiogenesis, we established a xenograft mouse model of ESCC to simulate the in vivo tumor environment. We injected exosomes into tumors and found that CAF‐derived exosomes not only promoted lymphangiogenesis in these tumors, but also promoted the growth of ESCC. These findings suggested that different components in CAF‐ and NF‐derived exosomes were responsible for their differential effects on lymphangiogenesis, which is essential for ESCC growth.

Hypoxia in the TME caused CAFs to secrete angiogenic factors that promote tumor angiogenesis and lymphangiogenesis.^33^, ^34^ In addition to the direct secretion of cytokines, CAFs also exert their actions through exosomes. Exosomes contain multiple components, including miRNAs,^35^, ^36^ which are negative regulators of gene expression. These miRNAs are involved in the occurrence, proliferation, metastasis, and invasion of multiple human malignancies. Exosomes can transmit information through miRNAs, allowing cell–cell communication, and thus play a crucial role in tumor progression.^37^, ^38^ In this study, we selected a high‐throughput RNA sequencing dataset of CAFs in ESCC from the GEO database to screen for miRNAs differentially expressed between NF‐ and CAF‐derived exosomes. Finally, we selected miR‐100‐5p as the target miRNA for further experiments. Therefore, in the present study, RNA was extracted from NF‐ and CAF‐derived exosomes. A marked reduction in miR‐100‐5p levels was noted in CAF‐derived exosomes, indicating that this reduction may mediate the lymphangiogenesis‐promoting effect of CAF‐derived exosomes. Many previous studies have reported that the dysregulation of miR‐100‐5p is involved in the occurrence, development, and drug resistance of tumors. Moreover, miR‐100‐5p plays different roles in different tumors. In some tumors, miR‐100‐5p can promote tumor progression as an oncogene, while in some tumors, miR‐100‐5p functions as a tumor suppressor.^39^, ^40^ Accordingly, lentiviral constructs were created, and the effect of miR‐100‐5p on lymphangiogenesis in ESCC was assessed. The miR‐100‐5p mimic effectively inhibited lymphangiogenesis in vivo and in vitro, whereas the miR‐100‐5p inhibitor promoted this process.

IGF1R, which regulates malignant behavior in a variety of tumors, is a downstream target of miR‐100‐5p.^41^, ^42^, ^43^ Interestingly, reports show that IGF1R is involved in the generation of blood vessels and lymphatic vessels in multiple tumors.^44^, ^45^ The downstream miR‐100‐5p targets predicted by online prediction software were similar to those reported previously. Then, we confirmed the relationship of miR‐100‐5p and IGF1R using the dual luciferase reporter and fluorescence in situ hybridization assay. Many studies have shown that IGF1R achieves its effect on tumors by activating the PI3K/AKT pathway.^46^, ^47^, ^48^ As a classical intracellular signaling pathway, the PI3K/AKT pathway is activated by insulin signaling and regulates cell growth, apoptosis, and metabolism.^49^, ^50^ Many studies have proved that inhibiting IGF1R and PI3K/AKT can effectively prevent the malignant biological behavior of ESCC, and improve the treatment sensitivity.^51^, ^52^, ^53^ Furthermore, this pathway has also been implicated in the generation of blood vessels and lymphatic vessels in a variety of tumors.^54^, ^55^ To verify whether the IGF1R/PI3K/AKT pathway mediates the regulation of ESCC lymphangiogenesis by miR‐100‐5p in CAF‐derived exosomes, we detected IGF1R, PI3K, AKT, and p‐AKT expression in TLECs and tumors. Western blotting revealed that IGF1R levels were decreased and the PI3K/AKT pathway was inhibited in TLECs and tumors from the miR‐100‐5p mimic groups. Moreover, the opposite effect was noted in TLECs and tumors from the miR‐100‐5p inhibitor group. These findings validated the inferences obtained from the previous experiments.

To our knowledge, this is the first study to demonstrate that CAF‐derived exosomes can promote lymphangiogenesis in ESCC and provide a preliminary exploration of the mechanism. Nevertheless, our study has certain limitations. miR‐100‐5p has countless downstream targets. In this study, we only focused on the most likely target, IGF1R. Whether other downstream targets of this miRNA are involved in ESCC lymphangiogenesis requires further investigation. Moreover, the complexity of cellular signaling pathways cannot be ignored. Therefore, other pathways besides the PI3K/AKT pathway may also regulate lymphangiogenesis, and these will be the focus of our future studies.

**FIGURE 8:**
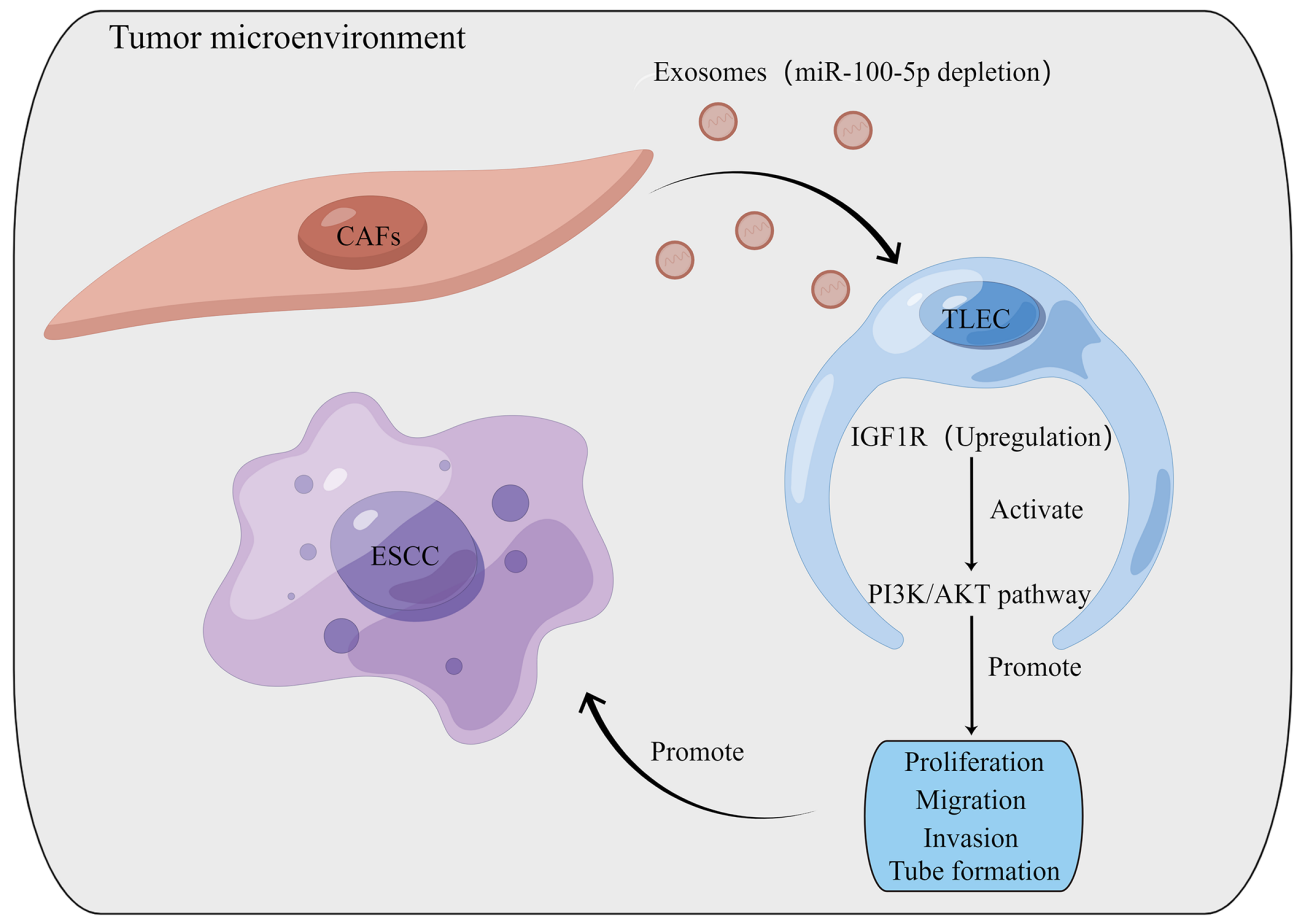
Diagram of the hypothetical mechanism clarifying the effect of miR‐100‐5p depletion in cancer‐associated fibroblast‐derived exosomes on lymphangiogenesis in esophageal squamous cell carcinoma.

In conclusion, the depletion of miR‐100‐5p in CAF‐derived exosomes increases IGF1R levels in TLECs, activating the PI3K/AKT pathway, which promotes lymphangiogenesis in ESCC, which affects ESCC progression (Figure 8). Therefore, miR‐100‐5p could be used as an exosome‐carried targeted drug that inhibits lymphangiogenesis and therefore suppresses lymphatic metastasis in ESCC. These findings could be used to develop strategies for the targeted treatment of ESCC.

## AUTHOR CONTRIBUTIONS

**Chao Chen:** Conceptualization (equal); data curation (equal); formal analysis (equal); methodology (equal); resources (equal); software (equal); visualization (equal); writing – original draft (equal); writing – review and editing (equal). **Chenbo Yang:** Formal analysis (supporting). **Xiangyu Tian:** Methodology (supporting). **Yinghao Liang:** Methodology (supporting). **Shuaiyuan Wang:** Formal analysis (supporting). **Xiaoqian Wang:** Software (supporting). **Yuwei Shou:** Resources (supporting). **Hui Li:** Software (supporting). **Qiankun Xiao:** Methodology (supporting). **Jiao Shu:** Formal analysis (supporting). **Miaomiao Sun:** Funding acquisition (equal); project administration (equal); validation (equal); writing – review and editing (equal). **Kuisheng Chen:** Funding acquisition (equal); project administration (equal); validation (equal); writing – review and editing (equal).

## CONFLICT OF INTEREST STATEMENT

The authors declare no conflicts of interest.

## Supporting information


Figure S1.

Figure S2.

## Data Availability

The article includes all data in the study, and further inquiries can be directed to the corresponding authors.
