# Supplementary figures and images for: Downregulation of miR‐100‐5p in cancer‐associated fibroblast‐derived exosomes facilitates lymphangiogenesis in esophageal squamous cell carcinoma
Source: Cancer Med. 2023 May 15;12(13):14468–83. doi: 10.1002/cam4.6078 (PMC10358253; doi:10.1002/cam4.6078)

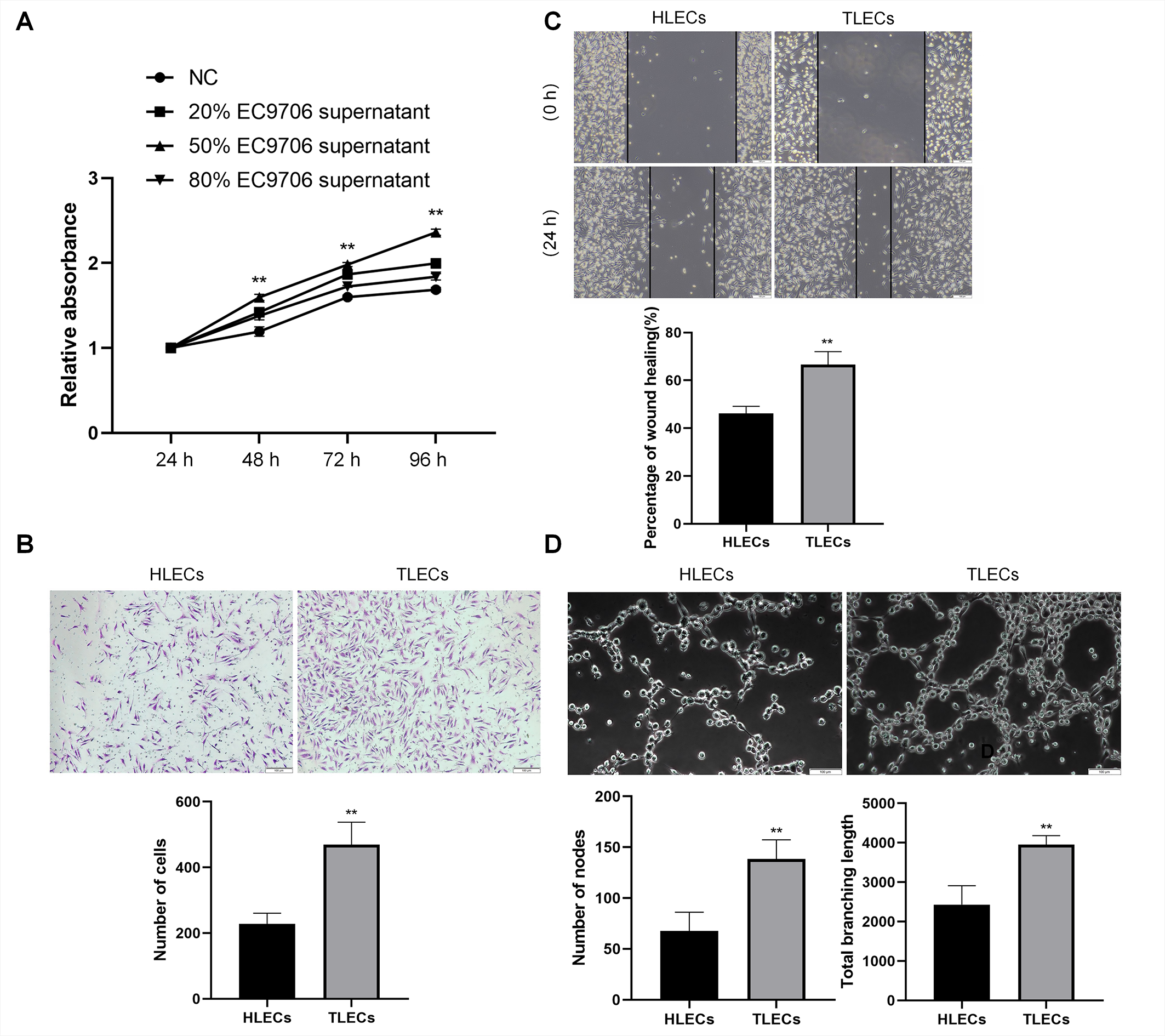

Supplement: Supplementary file 1 — Figure S1. [file CAM4-12-14468-s001.tif]

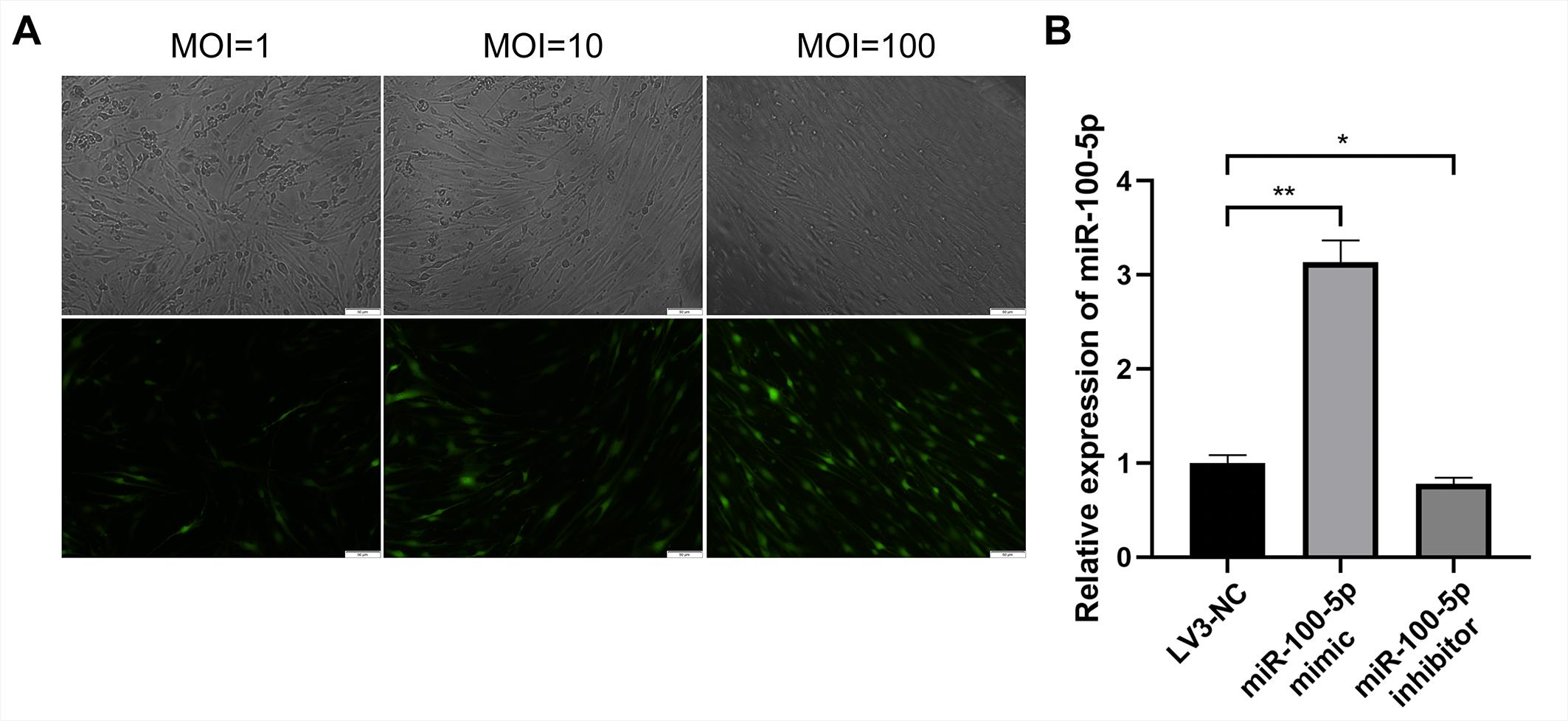

Supplement: Supplementary file 2 — Figure S2. [file CAM4-12-14468-s002.tif]
